# Benign intracranial hypertension associated to blood coagulation derangements

**DOI:** 10.1186/1477-9560-4-21

**Published:** 2006-12-24

**Authors:** Domenico De Lucia, Marisanta Napolitano, Pierpaolo Di Micco, Alferio Niglio, Andrea Fontanella, Giuseppe Di lorio

**Affiliations:** 1 Division of Pathology, Second University of Naples, Naples, Italy; 2 Internal Medicine Division, Buonconsiglio Fatebenefratelli Hospital of Naples, Naples, Italy; 3 Biochemistry and Biotechnology Department and Ceinge Scarl, "Federico II", University of Naples, Naples, Italy; 4 Division of Internal Medicine Second University of Naples, Naples, Italy; 5 Center for Migraine; Second University of Naples, Naples, Italy

## Abstract

**Background:**

Benign Intracranial Hypertension (BIH) may be caused, at least in part, by intracranial sinus thrombosis. Thrombosis is normally due to derangements in blood coagulation cascade which may predispose to abnormal clotting activation or deficiency in natural inhibitors' control. The aim of the study is to examine the strength of the association between risk factors for thrombosis and BIH.

**Patients and methods:**

The incidence of prothrombotic abnormalities among a randomly investigated cohort of 17 patients with BIH, was compared with 51 healthy subjects matched for sex, age, body mass index, height and social background.

**Results:**

The number of subjects with protein C deficiency was significantly higher in patients than in controls (3 vs 1, p < .001; Fisher Exact Test). Moderate to high titers of anticardiolipin antibodies (β2-Glycoprotein type I) were found in 8 out of 17 patients.

Increased plasma levels of prothrombin fragment 1+2, fibrinopeptide A (FPA), and PAI-1 were demonstrated in patients group (5.7 ± 1.15 nM vs 0.45 ± 0.35 nM; 8.7 ± 2.5 ng/mL vs 2.2 ± 1.25 ng/mL; 45.7 ± 12.5 ng/mL vs 8.5 ± 6.7 ng/mL, respectively; p < .001; Fisher Exact Test). Gene polymorphisms for factor V Leiden mutation, prothrombin mutation 20210 A/G, MTHFR 677 C/T, PAI-1 4G/5G, ACE I/D were detected in 13 patients.

**Discussion:**

In agreement with other authors our data suggest a state of hypercoagulability in BIH associated with gene polymorphisms. Our findings also showed that mutations in cardiovascular genes significantly discriminate subjects with a BIH history. The association between coagulation and gene derangements, usually regarded to as cryptogenic, may suggest a possible pathogenetic mechanism in BIH. So, a prothrombotic tendency may exist that would, at least in part, explain some cases of BIH.

Although based on a small population, these findings raise the exciting possibility of using these haemostatic factors as markers for selecting high-risk subjects in BIH disease.

## Background

Benign Intracranial Hypertension (BIH) is due to an increased intracranial pressure of unknown origin [1]. One of the possible causes of BIH may be due to intracranial venous sinus thrombosis [2], although cerebral angiograms could be normal in patients affected by BIH associated with conditions highly predisposing to venous thrombosis. This raises the possibility that unrecognised non-occlusive venous thrombus might impede cerebral spinal fluid (CSF) drainage [3].

Thrombosis is normally due to derangements in coagulation system which may predispose to abnormal clotting activation or to a deficient control of natural clotting inhibitors [4,5]. The risk of thrombosis is increased by factors that cause hypercoagulability or venous stasis, such as oral contraceptives, pregnancy or post-partum period, trauma, prolonged immobilization. However, the risk of thrombosis is also increased by hypercoagulable states due to inherited abnormalities of the coagulation system, such as factor V (FV) R506Q mutation, which causes resistance to activated protein C (PC) [6], prothrombin A20210G gene polymoprphism [7], and deficiencies of antithrombin III (AT III), PC or protein S (PS) [8]. Acquired abnormalities such as the presence of antiphospholipid antibodies can also induce an increased risk of thrombosis [9].

Increased plasma levels of the main inhibitor of fibrinolysis, plasminogen activator inhibitor type 1 (PAI-1), have been documented in subjects who subsequently developed myocardial infarction [10], while its association with venous thromboembolism is still matter of discussion.

The renin-angiotensin pathway plays a role in the regulation of PAI-1 plasma levels [11]. An insertion (I)/deletion (D) polymorphism of the angiotensin-convertying enzyme (ACE) gene has been related to plasma and cellular ACE levels [12]. Compared to the DD frequency in a control population, the frequency of the ACE DD genotype is higher in individuals with ischemic cardiovascular disease suggesting that ACE gene variant may contribute to the pathogenesis of this disease.

The C→T 677 transition in the methylen-thetrahydropholate reductase (MTHFR) gene [13] have widened the spectrum of inherited thrombophilia through hyperhomocysteinemia.

So an activation of blood coagulation may predispose to thrombin formation and fibrin deposition that may lead to thrombosis of large or small vessels.

In this study, we examined the strength of the association between risk factors for thrombosis and BIH disease.

## Patients and methods

### Patients

Seventeen unrelated patients with a documented diagnosis of idiopathic benign intracranial hypertension (BIH) according to World Health Organization criteria were studied retrospectively (4 men and 13 women; median age 31 years; range 15 to 55). They were referred to our thrombosis laboratory between February 1998 and May 1999 for a complete screening.

None of them had overt evidence of autoimmune or neoplastic disease. Subjects under anticoagulant or contraceptive treatment were previously excluded from the study.

The clinical records and the objective documentations of BIH were reviewed by two neurologists to confirm the diagnosis, based on clinical symptoms and signs of increased intracranial pressure (i.e. increased cerebrospinal fluid pressure, Computed Tomography and Nuclear Magnetic Resonance imagines with normal to small symmetrical ventricles).

### Healthy controls

Fifty-one healthy persons (30 men and 21 women; median age 32; range 19 to 52)_matched for sex, age, geographic origin, and level of education were enrolled as healthy controls in the study. They came from a population of biologically unrelated friends of the patients.

A thorough anamnesis was recorded for both patients and controls in order to know common risk factors for cardiovascular diseases (i.e. smoking, hypertension, diabetes, dyslipidemia, oral contraceptives use, personal and\or familial history of venous thromboembolism or cardio-cerebrovascular disease.

All subjects (i.e. patients and healthy controls) gave their written informed consent to the study.

### Laboratory tests

Blood samplings were performed for both cases and controls before 10.00 a.m. with subjects fasting and having rested for at least 20 minutes. Blood was collected in vacuum tubes contaning 3.8 percent (wt/vol) sodium citrate as anticoagulant. Plasma was then obtained by centrifugation at 2000 × g for 20 minutes, swap-frozen in liquid nitrogen and stored at -80°C within 2 h from collection. Blood samples in cases were collected between 6 and 24 months after the acute episode.

All cases were receiving secondary prophylaxis with either aspirin or ticlopidine, and none of them was on anticoagulant treatment with either warfarin or low molecular weight heparin. Laboratory measurements were subsequently performed in both cases and controls within 1 month from blood collection.

Prothrombin Time (PT) and Activated Partial Thromboplastin Time (APTT) were carried out according to Manifacturers recommendations. Fibrinogen (Fg) plasma levels were assayed by the Clauss functional method. All the measurements were determined on an automated coagulometer (ACL 2000, Instrumentation Laboratory, Milan).

Antithrombin III (ATIII) and Plasminogen (PLG) were determined by chromogenic methods (Chromogenix, Molndal; Sweden).

Antithrombin was further characterized by measuring antigen levels by immunoelectrophoresis with a policlonal antibody (Stago, Asmieres; France). Protein C (PC) activity was assayed by a clotting assay (Pro Clot, Instrumentation Laboratory, Milan; Italy). Protein C antigen levels were also measured by enzyme-linked immunosorbent assay (ELISA) with polyclonal antibodies (Dako, Glostrup; Denmark). Total protein S (total-PS) antigen was measured by ELISA with polyclonal antibodies (Dako). Free protein S (free-PS) was measured in the same way after precipitation of the C4 binding protein-protein S complex with polyethylene glycol 6000 (final concentration 3.5 percent) or directly by ELISA with a commercial kit that uses a specific monoclonal antibody (Asserachrom Free Protein S, Stago; France).

The diagnosis of poor response to anticoagulant action of activated protein C employed an APTT assay according to Dahlback and colleagues, with and without activated PC and in presence of an immunodepleted FV plasma (Instrumentation Laboratory; Milan) [14].

(Bouty, Milan; Italy) and Plasminogen Activator Inhibitor Type I (PAI-I) antigen levels were measured by ELISA (Bouty, Milan; Italy).

The measurements of prothrombin fragment 1+2 (F1+2), fibrinopeptide A (FPA) were assayed using an enzyme-linked immunosorbent test (ELISA method) (Enzygnost, Bhering, Scoppito, Italy), while Dimers-D (D-Dimer [s]) were tested with a specific kit (DILA HemosIL Dimertest Latex, Instrumental Laboratory, Milan, Italy).

DNA was extracted from peripheral blood using standard international procedures.

DNA analysis to identify a transition from guanine to adenine at position 1691 in the coagulation factor V gene was carried out as described by de Ronde and Bertina [15].

Screening for the MTMFR C->T 677 substitution was performed by amplification of a 198 bp DNA fragment followed by Hinf I digestion as described with modifications [13].

The PAI-1 4G/5G polymorphism was evaluated as previously reported [16].

The PCR technique, primers, and experimental conditions employed for the ACE genotyping were the ones suggested by Rigat et al. with some modifications [17].

### Statistical analysis

All the computations were perfomed according to the Statistical Package for Social Science (SPSS 6.1 for Macintosh) [18].

The frequencies of the alleles and genotypes among cases and controls were counted and compared by the chi-square test with the values predicted by assumption of the Hardy-Weinberg equilibrium. Chi-square analysis or Fisher exact-test were used to compare differences between discrete parameters. The differences between cases and controls were analysed by impaired Student's test for coagulation parameters, and by the Kruskall-Wallis test for age, according to their observed distribution. Multiple logistic analysis was performed by using LOGISTIC procedure for SPSS.

All the results are given as mean ± standard deviation (SD). A value of p < 0.05 was considered significant.

## Results

Clinical characteristics of studied subjects as a whole are shown in Table 1. Cases had the same age of healthy subjects; there was the same number of smokers among patients and controls and less often alcohol consumers (O.R. 0.55; 95% C.I., 0.21 to 0.65). Among subjects with pseudotumor cerebri there was a higher proportion of diabetics (O.R. 5.75; 95% C.I., 3.01 to 7.45), hypertensives (O.R. 8.11; 95% C.I., 3.40 to 18.15).

**Table 1:** Clinical Characteristics of patients and healthy subjects

	Patients (17)	Healthy Controls (51)
Men	4	30
Women	13	21
Age (IQR)	31 (15–55)	32 (19–51)

The two groups also differed for the number of hyperlipedemics (O.R. 6.25; 95% C.I., 4.05 to 8.72) and users of oral contraceptives (O.R. 5.15; 95% C.I., 3.65 to 7.21).

In addition, more frequently than controls, cases had an episode of venous thromboembolism (O.R. 7.60; 95% C.I., 3.50 to 16.05) and a family history of ischemic stroke (O.R. 9.15; 95% C.I., 6.75 to 17.05).

PT, APTT, and fibrinogen levels were similar among the two different groups of subjects, such as levels of plasminogen and t-PA.

No control or patient carried inherited abnormalities of antithrombin III or PS. The number of subjects with PC deficiency was significantly higher in patients than in controls (3 vs. 1 p < 0.001, Fisher Exact test).

Among controls only one subject showed a poor response to APC with FV gene R506Q mutation at heterozygous state (Table 2). Among cases, 2 individuals showed a poor response to anticoagulant action of APC (<0.75, which represents the cut-off point in our general population). One patient carried FV mutation R506Q at heterozygous state; the other subject did not show any mutation in FV gene, thus showing an acquired APC-resistance.

**Table 2:** Haemostatic parameters in pztients with BIH and controls. Values are expressed as main ± SD

	**Patients**	**Healthy Controls**	**P value**
PT(sec)	12 ± 0,5	11,5 ± 0,8	0.18, ns
aPTT (sec)	30 ± 2,5	31,5 ± 3,4	0.09, ns
Fibrinogen (mg/dl)	340 ± 80,7	360 ± 68,2	0.32, ns
ATIII (%)	72 ± 16,5	76 ± 14,7	0.34, ns
PG (%)	80 ± 14,7	74,5 ± 15,2	0.19, ns
t-PA(ng/ml)	6,8 ± 3,7	7,5 ± 4,8	0.58, ns
PAI-l(ng/ml)	45.7 ± 12.5	8.5 ± 6.7	**< 0.0001, s**
FPA (ng/ml)	8.7 ± 2.5	2.2 ± 1.25	**< 0.0001, s**
F1+2 (nM/l)	5.7 ± 1.15	0.45 ± 0.35	**0.0001, s**
D-dimers (ng/ml)	375 ± 65	225 ± 75	**< 0.0001, s**
PC(%)	64 ± 7.5	87 ± 14.5	**< 0.0001, s**
PS (%)	97 ± 23	103 ± 12	0.16, ns
Aβ_2_IgM (UM/l)	14.5 ± 6.8	0.38 ± 0.25	**< 0.0001, s**
Aβ_2_IgG(UG/l)	22.5 ± 11.5	0.58 ± 0.30	**< 0.0001, s**

Prothrombin Time: PT; Activated Partial Prothrombin Time: aPTT;

Antithrombin: AT III, Plasminogen: PG; tissue-Plasminogen Activator; t-PA, Plasminogen Activator Inhibitor type-1: PAI-1; Fibrinopeptide-A: FPA; Prothrombin Fragment 1+2: F1+2; Protein C activity: PC; Protein S activity: PS; IgM anticardiolipin antibodies Ig M and Ig G: Aβ_2_IgM and Aβ_2_IgG; s: significant; ns: not significant.

Moderate to high titers of IgG and IgM anticardiolipin antibodies (β2 GPI) were formed in 8 out of 17 patients (above the cut-off point of 10.5 U GPL/L and 9.5 U MPL/L, respectively). Among cases the mean values ± SD of Ig G β2 GPI and IG M β2 GPI were 22.5 ± 11.5 U GPL/mL and 14.5 ± 6.8 U MPL/mL respectively. Among controls the mean values ± SD of Ig G β2 GPI and Ig M β2 GPI were 0.58 ± 0.30 U GPL/mL and 0.38 ± 0.25 U MPL/mL, respectively (p < .001 and p < .001, Fisher Exact test).

Increased plasma levels of prothrombin fragment 1+2, FPA, D-Dimer(s) and PAI were demonstrated in patients group (5.7 ± 1.15 nM vs. 0.45 ± 0.35 nM, 8.7 ± 2.5 ng/mL vs. 2.2 ± 1.25 ng/mL; 375 ± 65 ng/mL vs. 225 ± 75 ng/mL; 45.7 ± 12.5 ng/mL vs 8.5 ± 6.7 ng/mL, respectively; Fisher Exact test).

As shown in Table 3, carriers of the FV R506Q mutation were 1 (6%) at heterozygous state among cases, and 1 (2%) at heterozygous state among controls. The O.R. associated with FV R506Q was 3.7 (95% C.I., 1.78 to 5.75 X^2 ^= 6.2, p = .01).

**Table 3:** Mutational state frequence for FV R506Q, PT G20210A, MTHFR C677T, PAI-1 4G/5G, ACE I/D genes in patients and healhty subjects (HS).

Gene coagulation mutations	Homozygous Wilde-type		Heterozygous		Homozygous Mutated	
	Patients (17)	HS (51)	Patients (17)	HS (51)	Patients (17)	HS (51)
FV R506Q	94%	98%	6%	2%	-	-
PT G20210A	94%	98%	6%	2%	-	-
MTHFR C677T	41%	57%	41%	31%	18%	12%
PAI-1 4G/5G	47%	30%	35%	51%	18%	19%
ACE I/D	30%	46%	47%	43%	23%	11%

*FV R506Q*: factor V Leiden gene polimorphism

*PT G20210A*: prothrombin G20210A gene polymorphism

*MTHFR C677T*: Methylenetetrahydrofolate reductase C677T gene polymorphism

*PAI-1 4G/5G*: plasminogen activator inihibitor 1 4G\5G gene polymorphism

*ACE I\D*: angiotensin converting enzyme insertion\deletion gene polymorphism

DNA analysis for the prothrombin A 20210 mutation demonstrated 1 heterozygous carrier (6 %, 95% C.I., 2.05 to 9.55; X^2 ^= 6.2, p = .012) among patients and 1 heterozygous carrier among healthy subjects (2 %, 95% C.I., 1.05 to 3.65). Among patients, 3 individuals (18 %, 95% C.I., 12.05 to 24.55; X^2 ^= 9.6, p = .002) were homozygous for the T allele of the MTHFR gene and 7 individuals (41 %, 95% C.I., 24.05 to 59.55; X^2 ^= 13.4, p = <.0001) were heterozygotes for the same defect vs 6 subjects (12 %, 95% C.I., 7.35 to 17.65) with homozygosity for the T allele of the MTHFR gene and 16 subjects with heterozigosity of control group (31 %, 95% C.I., 19.05 to 46.07).

Genotype analysis of the PAI-1 gene showed that 6 subjects were heterozygous for 4G/5G polymorphism among patients (35%, 95% C.I., 21.05 to 39.60; X^2 ^= 3.5, p = .06) and 3 subjects among patients showed homozygosity for 4G allele (18 %, 95% C.I., 12.25 to 23.15; X^2 ^= 5.03, p = .02), while 26 individuals were heterozygous for 4G/5G gene polymorphism (51 %, 95% C.I., 38.75 to 69.04) and 10 were homozygotes for 4G allele (19 %, 95% C.I., 11.85 to 24.14) among controls.

ACE gene I/D polymorphism was evidenciated in 8 patients at heterozygous state (I/D, 47 %, 95% C.I., 31.05 to 57.20; X^2 ^= 10.08, p = .001) and in 4 patients at homozygous state (D/D 23 %, 95% C.I., 14.15 to 33.45; X^2 ^= 15.55, p = <.0001) while 22 healthy subjects were heterozygotes for the same mutation (43 %, 95% C.I., 28.85 to 61.25) and 7 healthy subjects were homozygotes (11 %, 95% C.I., 2.05 to 9.55).

## Discussion

Abnormalities of the clotting system such as natural anticoagulant deficiencies, resistance to anticoagulant action of APC, derangements in fibrinolytic pathway or an hypercoagulable state have been recognized as clear risk factors for venous thromboembolism [8]. Their role, however, in BIH is still matter of discussion [19].

Recent findings have suggested that intracranial venous sinus thrombosis, favoured by thrombophilia/hypofibrinolysis, may cause BIH, determining an obstruction to cerebro-spinal fluid resorption-outflow [20].

On this topic, Sussmon et al. already reported that a state of hypercoagulability may exist in BIH disease [2].

Therefore, this state may also be associated with some mutations of the genes predisposing to hypercoagulability such as FV R506Q and FII G20210A and the C677T polymorphism in MTHFR gene and cardiovascular genes such as PAI-1 4G/5G and ACE gene I/D mutation. Moreover, also an association with antiphospholid antibodies has been found in our results so acquired thrombophilia should be considered; antiphospholipid syndrome in fact may be associated also to several impairments of central nervous system besides stroke [21].

Our results, throwing light on a compromised haemostatic balance equilibrium, provide new insights into the pathogenesis of BIH disease, which may be associated with high plasma levels in D-Dimer(s), PAI-1, F1+2 and FPA other than cytologic or chemical abnormalities of CSF that are able to contribute to the signs and symptoms of BIH.

The observation of hypercoagulable state and gene polymorphisms in a group of patients with BIH disease may also suggest the hypothesis of an association between coagulation derangements and venous thrombosis in BIH pathogenesis.

This hypothesis must be interpreted, however, with caution, because of the absence of objective instrumental findings in patients with BIH demonstrating the possibility that unrecognised non occlusive venous cerebral thrombus might impede CSF drainage as well as the high prevalence of gene polymorphisms predisposing to thrombophilia in the general healthy population. Furthermore, another limitation of our study may be find in the statistical model because the small number of enrolled patients compared with an increased of matched controls but this limit may be due to in part to the severe criteria that allow BIH diagnosis after a clinical suspect.

In conclusion, according to our findings the association of hypercoagulability based on biochemical or genetic abnormalities and BIH is probably not coincidental, in particular in subjects with inherited thrombophilia. So, our data may suggest that some cases of pseudotumor cerebri may recognise a thrombotic pathogenesis; although based on a small population, our results, in fact, seem to underline a clear association between hypercoagulable state, confirmed both with markers of hypercoagulability (i.e. fibrinopeptide A, prothrombin fragment 1+2 and D-Dimer) and with tests to look for inherited thrombophilia (i.e. factor V Leiden, prothrombin A20210G, PAI 4G/5G, ACE I/D, MTHFR C677T).

However, because based on a small population our data concerning the involvement of hypercoagulability in the development of BIH is intriguing and should be confirmed on large based randomized clinical trial because actually we have not chance to screen any type of asymptomatic subject potentially at risk of BIH.
